# Reduced field of view alters scanning behaviour

**DOI:** 10.1007/s10055-025-01125-0

**Published:** 2025-03-22

**Authors:** E. M. J. L. Postuma, F. W. Cornelissen, M. Pahlevan, J. Heutink, G. A. de Haan

**Affiliations:** 1 Department Clinical and Developmental Neuropsychology, Faculty of Behavioral and Social Sciences, University of Groningen, Groningen, The Netherlands; 2 Royal Dutch Visio, Centre of Expertise for Blind and Partially Sighted People, Huizen, The Netherlands; 3 Laboratory for Experimental Ophthalmology, University Medical Center Groningen, University of Groningen, Groningen, The Netherlands

**Keywords:** Field of view, Eye movements, Head movements, Mobility, Head mounted displays, Rehabilitation

## Abstract

**Introduction:**

Virtual reality environments presented through head mounted displays (HMDs) hold promise for training or studying mobility activities, such as cycling, walking, and street crossing. Yet, the limited field of view (FoV) of HMDs may influence scanning behaviour, reducing the translatability of findings to real-life situations. This study aims to (i) investigate how a reduced FoV influences scanning behaviour during mobility activities, and (ii) whether these alterations in scanning vary across these activities.

**Method:**

Sixteen participants performed a real-life walking, cycling and street crossing activity twice; once with and once without a reduced FoV. A mobile eye-tracker with a built in gyroscope recorded scanning behaviour. Scanning behaviour was evaluated in terms of saccadic frequency and amplitude, horizontal head movement frequency and amplitude, and the horizontal and vertical eye position.

**Results:**

The participants performed more horizontal head movements with larger amplitudes during the reduced FoV compared to the normal FoV. Additionally, they distributed their horizontal eye position more towards the central regions and less towards their peripheral regions. Overall, the range of both horizontal and vertical eye position decreased. The impact of the reduced FoV on horizontal head movement amplitude, horizontal eye position, and vertical eye position varied across activities.

**Conclusion:**

Generally, individuals seem to compensate for a reduced FoV by making more horizontal head movements with large amplitudes, while reducing the eye position distribution. Consequently, caution is advised when translating outcomes on scanning behaviour observed in HMDs to those expected in real-life situations.

**Supplementary Information:**

The online version contains supplementary material available at 10.1007/s10055-025-01125-0.

## Introduction

Virtual reality (VR), displayed through head mounted displays (HMDs), has emerged as a promising tool within rehabilitation and research, facilitating the training and studying of mobility activities, including cycling, walking and street crossing (Finley et al. 2021; Gestefeld et al. 2020; Nazemi et al. 2021; Pala et al. 2021; Sakhare et al. 2019; Schneider and Bengler 2020; van Paridon et al. 2021; Zeuwts et al. 2021). It offers considerable advantages over real-life settings, particularly the ability to construct safe, standardised and controllable environments (Clay et al. 2019). Moreover, VR enables interactive engagement with environments mimicking real-life situations, which may potentially enhance the translatability of training or research outcomes to real-world contexts. Nevertheless, the degree of this translatability is subject of ongoing debate (Schneider and Bengler 2020; Schwebel et al. 2008). For instance, scanning behaviour during mobility activities in VR displayed via HMDs can deviate from the scanning patterns observed in similar real-life situations (Berton et al. 2019, 2020; Drewes et al. 2021). Such differences pose a possible limitation for scientists and rehabilitation professionals when attempting to interpret VR outcomes.

Recent studies have observed differences in scanning behaviour between virtual environments compared to similar real-world settings during mobility activities when using HMDs (Berton et al. 2019, 2020; Drewes et al. 2021). Specifically, individuals produce fewer and shorter eye-movements, and show increased changes in head orientation when walking in a virtual environment compared to in the real-life situation (Berton et al. 2019, 2020; Drewes et al. 2021). This suggests that individuals tend to make more head movements and less eye movements during virtual compared to real-life walking. Interestingly, the coverage of fixation locations remained consistent between virtual and real environments (Berton et al. 2020). It is hypothesised that the disparities in scanning behaviour can be attributed to the restricted field of view (FoV) of HMDs, limiting the information obtainable through eye movements alone, as some information lies beyond the device’s edges (Berton et al. 2019, 2020). Nevertheless, the impact of the reduced FoV on scanning behaviour has yet to be determined. Furthermore, considering the apparent task-specificity of scanning behaviour (Foulsham 2015), the impact of the limited FoV likely varies across different mobility activities. For instance, head movements may be more crucial for safe street crossing compared to navigating a crowded area, which may affect the effect of the restricted FoV. To understand such influences, it is imperative to selectively examine the effect of a reduced FoV on scanning behaviour during various mobility activities.

In this study, we aim to examine (i) the effect of a reduced FoV, as is present in HMDs, on visual scanning behaviour during three different mobility activities: cycling, walking and street crossing. Second (ii), we studied whether this effect differs between these three activities. Participants will perform these mobility activities in real-life situations, both with and without a limited FoV, while their scanning behaviour is recorded. Following previous investigations, we anticipate that individuals will perform more and larger head movements while reducing the amplitude and frequency of eye movements in the presence of a reduced FoV. In turn, we expect this to lead to fewer and shorter saccades, whereas the number and amplitude of head movements increases. Additionally, we expect that the impact of the reduced FoV on scanning behaviour differs between the mobility activities as scanning demands are task-specific (Foulsham 2015). The findings of this study will enhance our understanding of disparities between scanning behaviour in VR environments displayed by HMDs and scanning behaviour in real-life situations. This step is crucial for correct interpretation of scanning behaviour when wearing HMDs, particularly when this will be used to make recommendations for real-life situations.

## Method

### Participants

Sixteen participants were recruited among students of the University of Groningen. All participants were ≥ 18 years old, reported no psychological, neurological or motor impairments, and provided informed consent. Individuals with corrected visual acuity were excluded if they wore glasses and were unable to wear contact lenses. The participants were divided into two groups, with females alternately assigned to group A and B, based on the order of inclusion. The same process was applied to males, ensuring an equal gender distribution. The two groups performed the experimental mobility activities in a different order. The study was approved by the ethical committee of the Psychology faculty of the University of Groningen (Research code: PSY-2122-S-0375). The demographic characteristics of the participants are presented in Table 1.

**Table 1:** Demographics participants

	Group A (*N* = 8)	Group B (*N* = 8)	Total (*N* = 16)
Gender (N Female/N Male)	3/5	3/5	6/10
Age (yrs) (M (SD) [range])	22.8 (1.3) [21–25]	23.5 (1.1) [22–25]	23.1 (1.2) [21–25]

### Apparatus

The Pupil Invisible eye-tracker (Pupil Labs, Berlin, Germany) was used to record scanning behaviour. This device consists of two eye cameras and a scene camera with an FoV of 82 × 82 degrees utilised for eye-position estimation in an eye-in-head reference frame at a sample frequency of 200 Hz. The eye-tracker is automatically calibrated by a deep learning-based eye position estimation pipeline and is proposed to have an accuracy of 4° with a lower accuracy in the peripheral areas (Tonsen et al. 2020). While the scene camera has an FoV of 82 × 82 degrees, the eye-tracker was able to track the eye position outside of this reference frame. The device also contains an inertial measurement unit (i.e. accelerometer and gyroscope) utilised to measure head rotation and acceleration at a sample frequency of 200 Hz. The tool designed to reduce the FoV was constructed using sturdy cardboard material, featuring both a horizontal and vertical angle of 90°, mirroring the typical binocular FoV of current HMD devices. An elastic cord was attached to the device, allowing it to be securely and firmly mounted on each participants’ head.

### Protocol

**Fig. 1:**
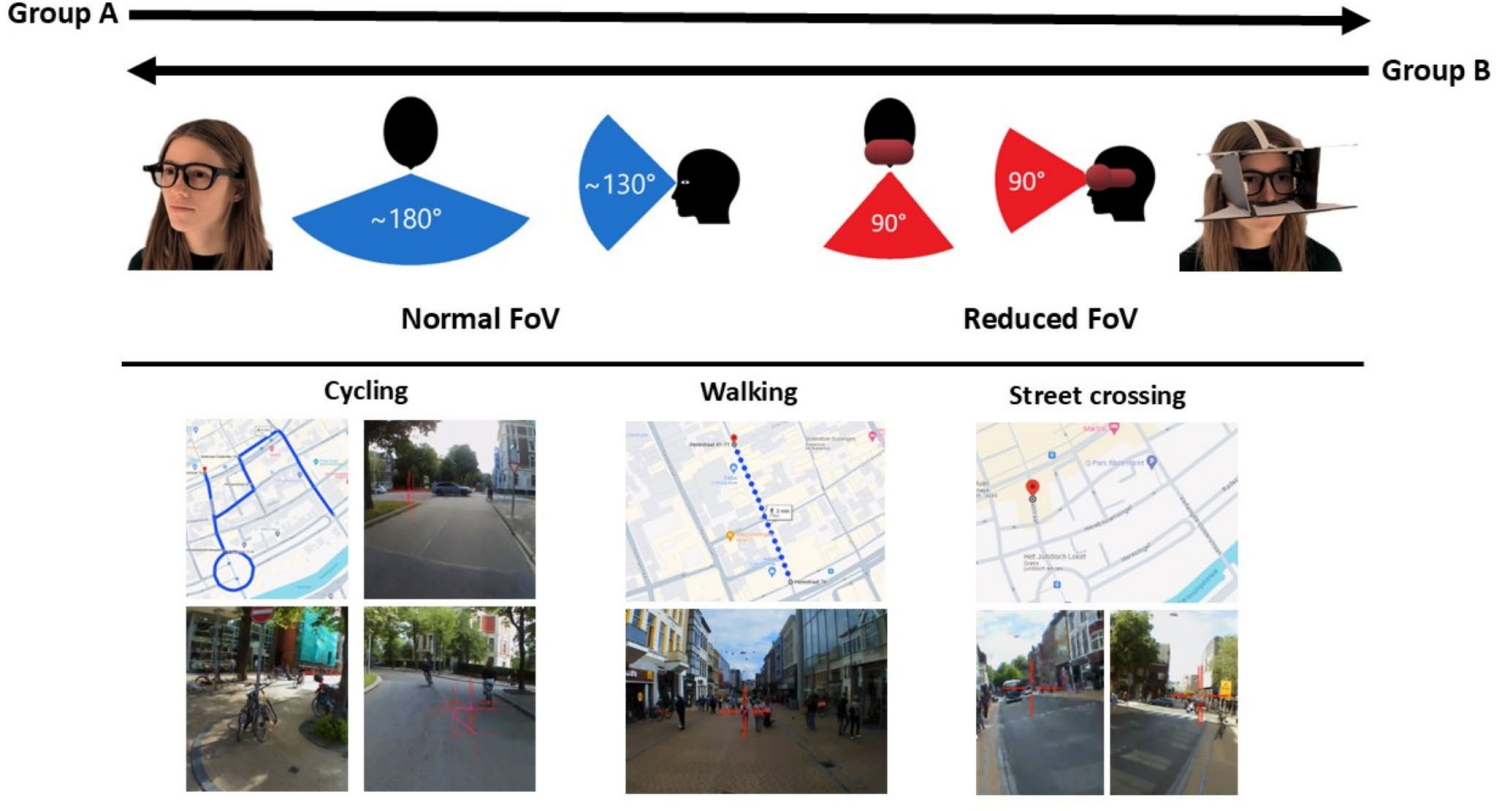
Protocol of the experiment. Two groups (Group A and B) performed the activities, in the order of cycling, walking and street crossing, twice, group A starting with the normal FoV condition and group B starting with the reduced FoV condition. The cycling route, walking route and street crossing location area presented in the lower panel together with illustrations of the activity from the participant perspective

The experiment was conducted in the city centre of Groningen, the Netherlands, during daytime on working days. The two participant groups performed the mobility activities twice, following the order of cycling, walking and street crossing (see Fig. 1). Group A initially performed the three mobility activities with a normal (unrestricted) FoV, followed by the reduced FoV. Conversely, group B started with the reduced FoV and then switched to the normal FoV. Scanning behaviour was captured throughout all activities using the eye-tracker. Calibration validations of the eye-tracker were performed at the start of the experiment, after having performed the three activities once and at the end of the experiment. For this purpose, a research assistant held a QR code approximately 1 m in front of the participant. This QR code was slowly moved towards four distinct locations, while the participants tracked it with their eyes, keeping their heads fixed. The four distinct locations were roughly 10° to the right and above, 10° to the left and above, and 5 to the right and below, and 5° to the left and below the centre of the eye-tracker.

In the cycling activity, the participants were instructed to adhere to the traffic regulations as they cycled a predetermined route through the city centre of Groningen, lasting approximately 6 min. This route included a roundabout and various intersections, comprising both car lanes, bicycle lanes and mixed car and bicycle lanes. Prior to starting the cycling activity, the participants were informed of the cycling route. During the cycling activity, a researcher following the participant’s provided the specific directions. Note, that all participants were familiar with cycling in the city centre of Groningen. In the walking activity, the participants were instructed to walk through a shopping street for approximately 4 min, reversing direction midway and retracing their steps. Lastly, the participants crossed a street eight times, which was accessible to both motorised vehicles and cyclists. They were instructed to wait until at least one road-user had passed the crossing location, and then cross the street when they deemed it was safe. This prevented participants from crossing during periods without any traffic present. Throughout each activity, a researcher followed the participants and could intervene in case of potential unsafe situations. This researcher also provided the directions during the cycling activity. Importantly, the cycling and walking route as well as the street crossing location, remained identical across all participants and FoV conditions.

**Fig. 2:**
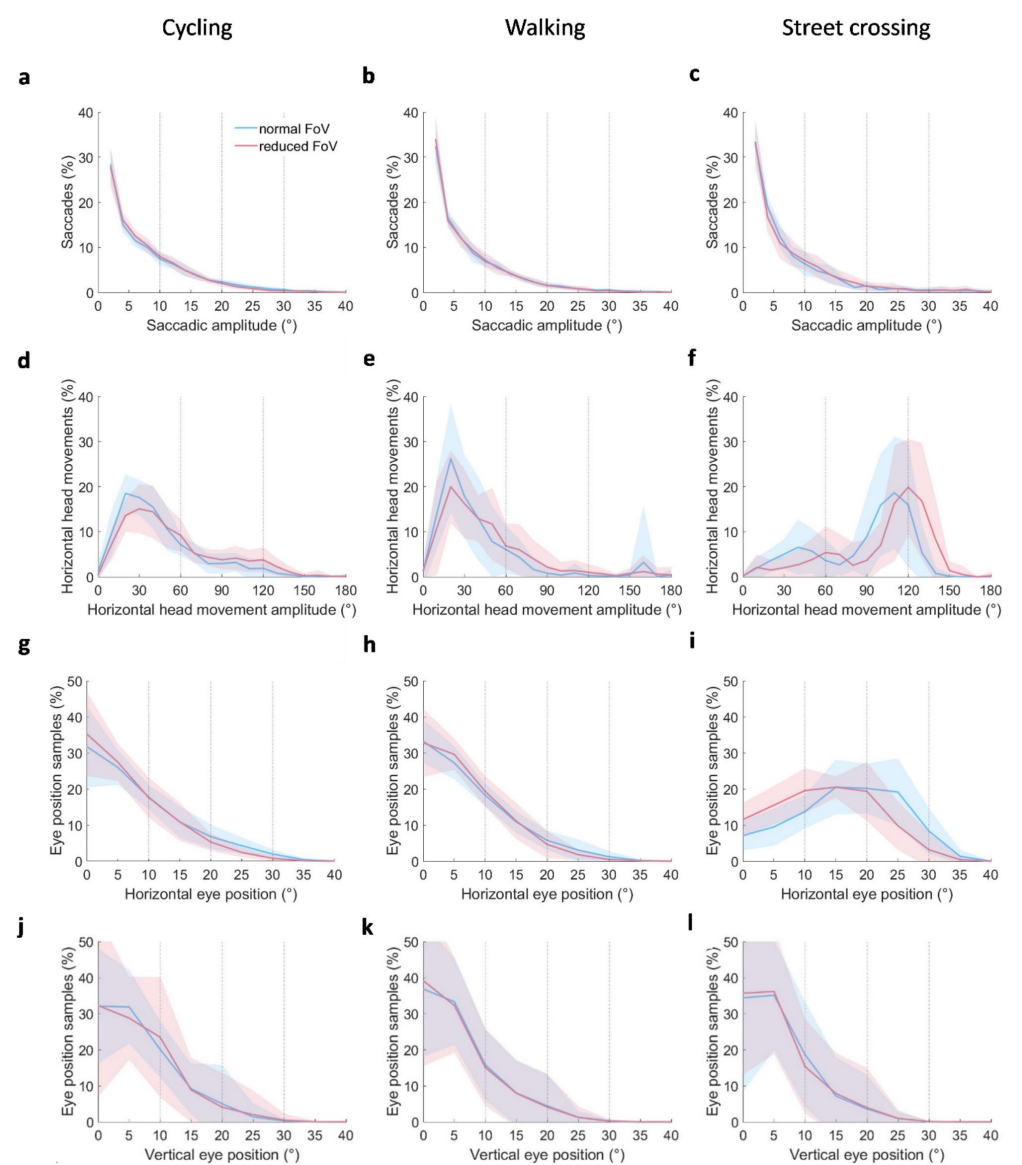
Distributions of saccadic amplitude (**a**–**c**), horizontal head movement amplitude (**d**–**f**), horizontal eye position (**g**–**i**) and vertical eye position (**j**–**l**) for the reduce FoV (r-FoV) condition presented in red and the normal FoV condition (n-FoV) presented in blue per activity: Cycling (left panel), Walking (middle panel), Street crossing (right panel). Vertical blue and red lines represent the standard deviations of the distribution plots for the n-FoV condition and the r-FoV condition respectively. The thin black dotted vertical lines indicate the ranges used to statistically analyze the effect of a reduced FoV on the distributions of eye and head movement parameters. The average distribution of the eye and head movement parameters within these ranges are illustrated in Fig. 5 in the appendix

### Data analysis

The eye-tracking and horizontal head rotation (i.e. gyroscope) data was analysed by using the pupil player software (v3.5.1, Pupil Labs, Berlin, Germany) and MATLAB (v2022a, Mathworks, Massachusetts, United States). The recorded eye-tracking and horizontal head rotation data were trimmed to encompass intervals corresponding to the onset and offset of the activities by using pupil player software (Pupil Labs, Berlin, Germany). Subsequently, the estimated eye-position and horizontal head rotation data was analysed by using MATLAB. The eye-position data was provided by the pupil labs software in distorted pixel frames in an eye-in-head reference frame. We undistorted the data by using the camera matrix of the scene camera and converted the data into degrees. Next, we removed data points from the dataset that were classified as blinks by the blink detection algorithm provided by Pupil Labs (v1.0.8., Berlin, Germany).

To evaluate scanning behaviour we firstly classified saccades from the eye-position data and horizontal head movements from the horizontal head rotation data (i.e. rotation measured by the gyroscope on the yaw axis). Saccades, defined as fast eye-movements altering the visual image on the retina, were classified by a slow and fast eye movement classifier with a variable velocity threshold (Hooge and Camps 2013). This approach encompasses both fixations and smooth pursuits as slow eye-movement events. Therefore, it distinguishes eye-movement events into events that alter the visual image on the fovea (i.e. saccades) and events that maintain a stable foveal image (i.e. fixations and smooth pursuit). Moreover, this approach has the advantage of reducing the number of saccades misclassified as noise due to bodily movements, which could be particularly important in dynamic activities such as cycling and walking. The shortcomings are, however, that we are not able to distinguish between vestibular ocular reflexes and saccades, and that during phases with high noise ratio, small saccades may go undetected. Saccadic initiation and termination points were classified by detecting the time points where the eye velocity crossed the variable threshold. We excluded saccades with an amplitude below 1 degree, and saccades that were also classified as blinks. Additionally, we excluded slow eye movement phases (i.e. fixations and smooth pursuit) with a duration less than 0.060 s. Horizontal head movements were classified by detecting velocity peaks above 50°/s, flanked by preceding and succeeding valleys with velocities of less than 25°/s. The horizontal head movement initiation and termination time points were identified by detecting the nearest valleys below a rotation of 25 °/s surrounding the selected velocity peaks. These thresholds were based on a test we conducted prior to application. Six individuals were instructed to walk and cycle along a street while alternately performing no head movements and several deliberate head movements. As a result, our head movement data includes only movements intended to alter visual input, excluding movements linked to walking or cycling. A limitation of this approach is the potential to miss small head movements that may still influence visual input.

In this study, we analysed how the reduced FoV influences the distribution of saccadic amplitude, horizontal head movement amplitude, horizontal eye position and vertical eye position. Additionally, we examined its overall effect on the saccadic frequency and amplitude, the horizontal head movement frequency and amplitude, and the horizontal and vertical eye position interquartile range.

The saccadic amplitude was calculated by using the Pythagoras theorem on the difference in both x and y coordinates (in degrees) between the saccadic initiation and termination points. The horizontal head movement amplitude was calculated by integrating the horizontal head rotation data in °/s between the head movement initiation and termination time points. For the saccadic amplitude and horizontal head movement amplitude we utilised absolute values, as this study did not aim to investigate differences in direction. The horizontal and vertical eye position ranged from − 40 to 40°, with negative values indicating the leftward or downwards position and positive values indicating rightward or upward position. To focus on central versus peripheral position rather than left versus right or up versus down, we used the absolute position value ranging from 0 to 40°. Values closer to 0° denote a more centrally directed position, and values closer to 40° denote a more peripherally directed position. The original distribution plots with a position from − 40 to 40° can be found in the appendix Fig. 5.

To analyse the distributions, we plotted the percentage of saccades per saccadic amplitude, the percentage of horizontal head movements per head movement amplitude, and the percentage of eye position samples per horizontal or vertical eye position. For saccadic amplitude, horizontal and vertical eye position, we used bins of 2° ranging from 0 to 40°, whereas for horizontal head movement amplitude we used bins of 10° ranging from 0 to 180°. Additionally, we divided the distribution plots into ranges. The distribution plots for saccadic amplitude, horizontal and vertical eye position were divided into four ranges of 10° each within a range of 0 to 40°. The horizontal head movement amplitude distribution plots were divided into three ranges of 60° each within a range of 0 to 180°.

To examine the impact of the reduced FoV on the scanning behaviour overall, we analysed the saccadic frequency and mean amplitude, the horizontal head movement frequency and mean amplitude, and the horizontal and vertical eye position interquartile range. The saccadic and horizontal head movement frequency (in Hz) were calculated by dividing the number of classified saccades or horizontal head movements performed within each activity by the activity duration. The mean saccadic and horizontal head movement amplitude were computed by averaging all saccadic or horizontal head movements amplitudes performed within a mobility activity. Lastly, the interquartile range was calculated for the horizontal and vertical eye position with the original range of -40 to 40°. It is important to note that saccadic and eye position variables are described within an eye-in-head reference frame.

### Statistical analysis

Statistical analysis was performed in IBM SPSS Statistics (version 27, IBM, New York). A repeated measures ANOVA was conducted to examine the effect of the reduced FoV on the distribution of saccadic amplitude, horizontal head movement amplitude, horizontal and vertical eye position. The within-group factors included the FoV conditions (i.e. reduced vs. normal), the activities (i.e. cycling, walking, street crossing) and the ranges of the distribution plots: 0–10°, 10–20°, 20–30°, 30–40° for the saccadic amplitude, horizontal and vertical eye position, and 0–60°, 60–120°, 120–180° for the horizontal head movement amplitude. Additionally, the order of FoV conditions (i.e. group A vs. group B) was included as a between-group factor. In case of significant an FoV*Range interaction effect or an FoV*Range*Activity interaction effect, post hoc tests were conducted analysing the differences in distribution between the FoV conditions within each range and per activity. Additionally, post hoc tests were performed following significant FoV*Range*Order interaction effects, to analyse differences in distribution due to order within each range and per FoV condition.

To assess the impact of the reduced FoV on the overall scanning behaviour, a repeated measures ANOVA was performed with saccadic frequency and mean amplitude, horizontal head movement frequency and mean amplitude, and the horizontal and vertical eye position interquartile range as dependent variables. Within-group factors included the FoV conditions and the activities, while the order served as a between-group factor. Post-hoc-test were conducted following a significant FoV main-effect or a significant FoV*Activity interaction effects, to analyse the differences in scanning behaviour between the FoV conditions per activity. The alpha was set 0.05. Effect sizes were calculated using the partial eta squared, with values of 0.01, 0.06, and 0.14 indicating a small, medium and large effect size respectively.

## Results

### The influence of a reduced field of view on the distribution of scanning parameters

Figure 2 illustrates the distribution of saccadic amplitudes, horizontal head movement amplitudes, horizontal eye position and vertical eye position across the normal and reduced FoV condition for each activity. Figure 5 in the appendix further details these distributions by dividing them into three or four distinct ranges, allowing for clear visualisation and comparison of the effect of the reduced FoV on the distributions for each activity.

**Fig. 3:**
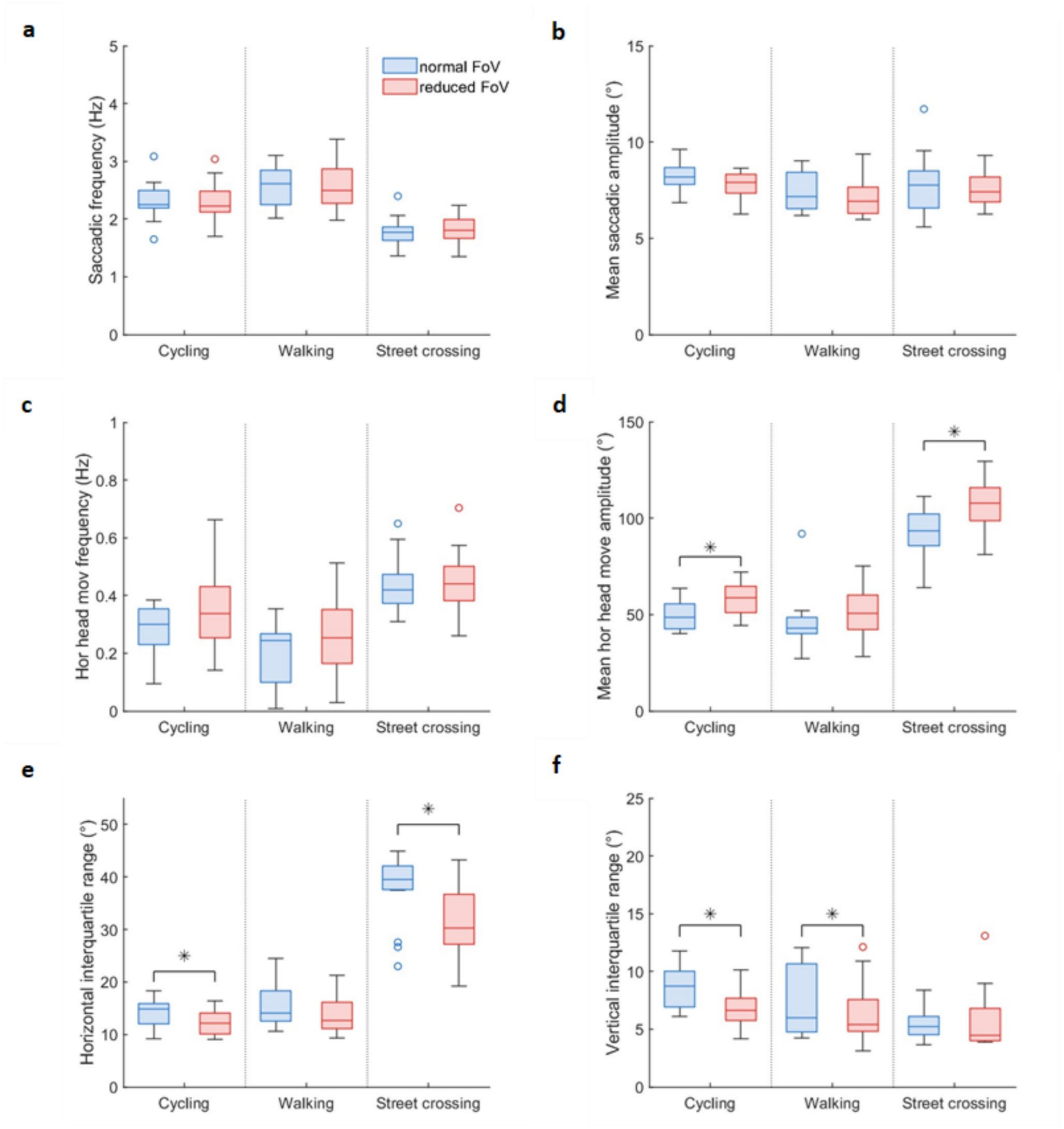
The saccadic behaviour during the normal- (blue) and reduced-FoV condition (red) for each activity (i.e. cycling, walking, street crossing) in terms of saccadic frequency (**a**) and mean amplitude (**b**), horizontal head movement frequency (Hor head move frequency) (**c**) and mean horizontal head movement amplitude (Mean hor head move amplitude) (**d**), and the horizontal (**e**) and vertical (**f**) eye position interquartile range

The reduced FoV influenced the distribution of horizontal head movement amplitude and horizontal eye position, with the extent of this effect varying between the three activities. This is indicated by a significant FoV*Range interaction-effect and FoV*Range*Activity interaction-effect, both of which have large effect-sizes (Table 2).

**Table 2:** Statistical analysis on the influence of the reduced FoV on the distribution of the scanning parameters

	FoV*Range^a^			FoV*Range*Activity^b^			FoV*Range*Order^c^		
	F(3,36)	p	η^2^	F(6,72)	p	η^2^	F(3,36)	p	η^2^
Saccadic amplitude distribution	0.671	0.478	0.053	1.81	0.182	0.131	0.520	0.900	0.004
Horizontal head movement amplitude distribution	**18.6**	**< 0.001**	0.570*	**10.4**	**< 0.001**	0.426*	0.028	0.973	0.002
Horizontal eye position distribution	**10.2**	**< 0.001**	0.459*	**4.32**	**0.006**	0.265*	358	0.784	0.029
Vertical eye position distribution	0.017	0.916	0.001	1.06	0.364	0.081	2.02	0.179	144*

Significant results in Bold

*Large effect-size

^a^Indicates whether the distribution of the parameters significantly differ between the FoV conditions within the defined ranges (0–10°, 10–20°, 20–30°, 30–40° for the saccadic amplitude, horizontal and vertical eye position, and 0–60°, 60–120°, 120–180°)

^b^Indicate whether the differences in distribution due to the FoV significantly vary between activity

^c^Indicate whether the order in which the activities were performed, whether starting with the normal or reduced FoV, influenced the effect of the reduced FoV on the distribution of the scanning parameters

**Fig. 5:**
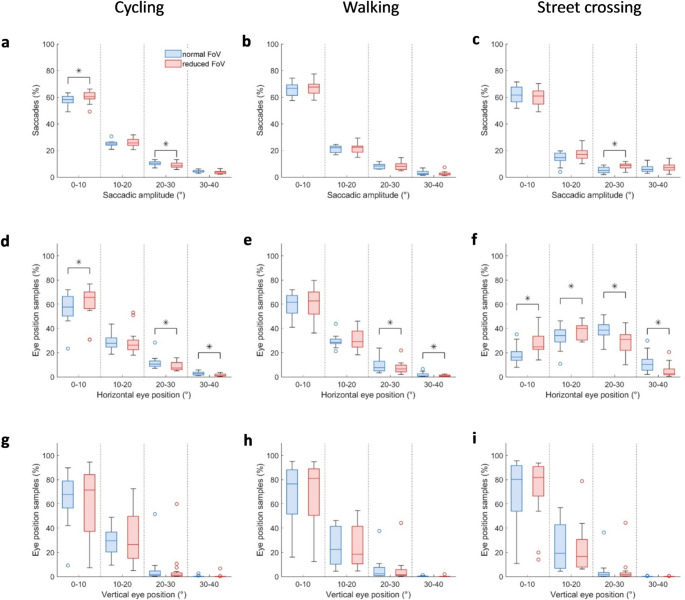
Box Plots illustrating the distributions per range of the parameters saccadic amplitude (**a**–**c**), horizontal head movement amplitude (**d**–**f**), horizontal eye position (**g**–*i*) and vertical eye position (**j**–**l**) for the reduced FoV (r-FoV) condition presented in red and the normal FoV condition (n-FoV) presented in blue per activity; Cycling (left panel), Walking (middle panel), Street crossing (right panel). The vertical lines represent the boundaries of the ranges

The reduced FoV resulted in fewer horizontal head movements with short amplitudes, and more horizontal head movements with long amplitudes (Fig. 2d–f). Specifically, while cycling, there was a reduction in horizontal head movements with amplitudes between 0–60° (*p* < 0.001) and an increase in those between 60–180° (*p* < 0.01) due to the reduced FoV (Fig. 2d). A similar pattern was observed during walking, although the impact of the reduced FoV was less pronounced. Specifically, individuals performed more horizontal head movements with an amplitude of 60–120° (*p* < 0.05) due to the reduced FoV  (Fig. 2e). The absence of a significant post hoc test in the other ranges may be partly related to one individual performing solely two head movements during the walking activity in the normal FoV condition, dividing the distribution of head movements equally (50%) between 0–60° and 60–120° (see also Fig. 5e in the appendix)). Figure 7A in the Appendix illustrate the distribution plot and analysis of horizontal head movement amplitudes during walking without this outlier, showing a reduction in horizontal head movements with an amplitude between 0–60° (*p* < 0.05), an increase in horizontal head movements between 120–180° (*p* < 0.05), and no difference in head movements with an amplitude between 60–120° due to the reduced FoV. During street crossing, the reduced FoV condition resulted in fewer horizontal head movements with amplitudes between 0-120° (*p* < 0.01) and more horizontal head movements with amplitudes between 120°-180° (*p* < 0.001) compared to the normal FoV condition  (Fig. 2f).

Across all three activities, the horizontal eye position was less often directed towards the peripheral regions during the reduced FoV condition compared to the normal FoV condition (*p* < 0.05; Fig. 2g–i). Additionally, the reduced FoV led to an increased central bias between 0–10° during cycling (*p* < 0.05) and 0–20° during street crossing (*p* < 0.05; Fig. 2g and i). This shift towards a more centrally directed eye position was not observed during walking (*p* > 0.45; Fig. 2h). It should be noted that the differences in distribution of horizontal eye positions between the FoV conditions during cycling and walking were small (≤ 4.7°).

**Fig. 6:**
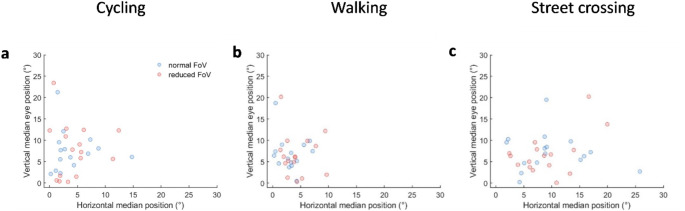
The median horizontal and vertical eye position (**a**–**c**) for the reduced FoV (r-FoV) condition presented in red and the normal FoV condition (n-FoV) presented in blue per activity; Cycling (left panel), Walking (middle panel), Street crossing (right panel)

The reduced FoV did not alter the distribution of saccadic amplitudes or vertical eye position, as indicated by the absence of an FoV*Range interaction effect, an FoV*Range*Activity interaction effect, and a lack of large effect-sizes (Table 2; Fig. 2a–c and j–k). Notably, Fig. 2j–k shows a high variability in the distribution of vertical eye position. Coincidingly, there was much variation in the median vertical eye position (ranges of 0.3–23.4° for cycling, 0.3–20.3° for walking, and 0.1–20.3° for street crossing), especially compared to the median horizontal (ranges of 0.0–14.7° for cycling, 0.3–9.7° for walking, and 1.9–25.8° for street crossing), as is illustrated in Fig. 6 in the Appendix.

**Table 3:** Statistical analysis of the influence of FoV on the average scanning parameters

	FoV^a^			FoV *Activitv^b^			FoV*Order^c^		
	F (1,12)	p	η^2^	F (2,24)	p	η^2^	F (3,36)	p	η^2^
Saccadic frequency (Hz)	0.009	0.927	0.001	0.245	0.785	0.020	3.19	0.099	0.210
Mean saccadic amplitude (°)	3.85	0.073	0.243*	1.03	0.372	0.79	0.000	0.994	0.000
Horizontal head movement frequency (Hz)	**5.53**	**0.037**	0.315*	0.34	0.967	0.003	0.441	0.529	0.035
Mean Horizontal head movement frequency (°)	**55.8**	**< 0.001**	0.823*	3.03	0.067	0.202*	1.40	2.59	0.105
Horizontal eye position interquartile range (°)	**17.1**	**0.001**	0.588*	3.33	0.086	0.217*	0.120	0.753	0.010
Vertical eye position interquartile range (°)	5.37	0.039	0.309	4.49	0.022	0.272	0.317	0.584	0.26

Significant results in Bold

*Large effect size

^a^Indicates whether the average scanning parameters significantly differ between the FoV conditions

^b^Indicate whether the differences on the average scanning parameters due to the FoV significantly vary between activity

^c^Indicate whether the order in which the activities were performed, whether starting with the normal or reduced FoV, influenced the effect of the reduced FoV on the average scanning parameters

### The influence of a reduced field of view on the scanning parameters overall

**Fig. 7:**
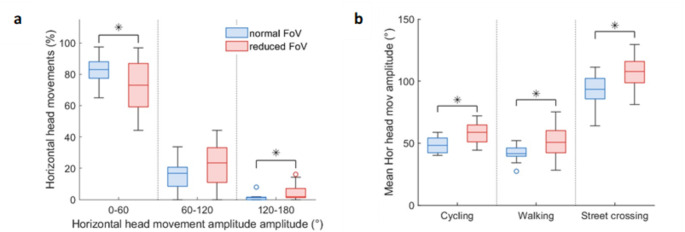
**a** The distribution of head movement amplitudes for the activity walking and **b** the mean horizontal head movement amplitude per activity during the normal- (blue) and reduced-FoV condition (red) without the outlier

Figure 3 shows the saccadic frequency and mean amplitude, the horizontal head movement frequency and mean amplitude, and the horizontal and vertical interquartile range in eye position for both reduced and normal FoV conditions across each activity. Overall, the reduced FoV condition led to an increase in horizontal head movement amplitude, while decreasing the horizontal and vertical eye position interquartile range compared to the normal FoV. This is indicated by significant FoV main-effects with large effect-size (Table 3 Fig. 3c and d). Additionally, the reduced FoV resulted in a lower horizontal head movement frequency overall (i.e. a significant FoV main-effect with large effect-size, Table 3), yet analysing the activities separately did not reveal differences between the two FoV conditions (*p* > 0.09). The increase in horizontal head movement amplitude and decrease in the horizontal eye position interquartile range was less present during walking. This is supported by an FoV*Activity interaction effect or an FoV*Activity comparison with a large effect size (Table 3) and a non-significant post hoc test for the activity walking (*p* > 0.075;  Fig. 3d and e). The absence of a significant post hoc test for the mean horizontal head movement amplitudes may be partly related to one individual performing solely two head movements during the walking activity in the normal FoV condition, one with an amplitude between 0–60° and one with an amplitude between 120–180°. This individual also showed a relatively high mean horizontal head movement amplitude (92°; Fig. 3d). Figure 7b in the Appendix illustrate the plot and analysis of mean horizontal head movement amplitude without this outlier, showing an increase in mean horizontal head movement amplitude due to the reduced FoV (*p* < 0.05).

In contrast, the decrease in the vertical eye position interquartile range was less present during street crossing (i.e. an FoV*Activity interaction effect with a large effect size, see Table 3, and a non-significant post hoc test for the activity street crossing, *p* > 0.9). The increase in horizontal head movement frequency due to the reduced FoV was consistent across the three activities (i.e. no FoV*Activity interaction effect and a lack of large effect sizes, Table 3). Additionally, the reduced FoV did not influence the saccadic frequency (i.e. no FoV main-effect, FoV*Activity interaction and a lack of large effect sizes). There was a trend towards a slight increase in saccadic amplitude during the reduced FoV condition compared to the normal FoV condition, indicated by a large effect size for the FoV main comparison. However, this difference in saccadic amplitude is negligible, approximately 0.34°.

### The effect of order

The order in which the activities were performed, whether starting with the normal or reduced FoV, did not influence the effect of the reduced FoV on the distribution of the scanning parameters, i.e. saccadic amplitude, horizontal head movement amplitude, horizontal eye position and vertical eye position (i.e. no FoV*Range*Order interaction effects; Table 2). The order also did not the effect of the reduced FoV on the scanning parameters overall (i.e. no FoV*Order interaction effects; Table 3).

### Data quality

On average, the participants took 5.4 min to finish the cycling route, 4.2 min to conclude walking activity, and 1.5 min to complete the eight street crossings. Two recordings failed to capture the eye and head movement data due to technical issues: one recording of the cycling activity under the normal field condition by an individual in group B and one recording of the walking activity under the normal field condition by an individual included in group A. Additional data loss consisted of time frames classified as blinks, which were removed. On average, the data loss per participant was 22.0% for cycling, 16.6% for walking, 25.9% for street crossing, and 21.5% across all three activities. The calibration validation determined a mean accuracy of 5.2° (SD = 1.6°), ranging from 2.4° to 8.7°. Detailed information on the data quality is provided in the Appendix Tables 5 and 6.

## Discussion

In this study, we examined (i) the impact of a reduced FoV, as presented in HMDs, on visual scanning behaviour during the mobility activities street crossing, walking and cycling. Our findings reveal that with a reduced FoV, individuals make more horizontal head movements with larger amplitudes, accompanied by a reduction in horizontal eye position towards peripheral regions. Additionally, the overall range in both horizontal and vertical eye position decreases with a reduced FoV, while the mean horizontal head movement amplitude increases accompanied by a slight increase in the horizontal head movement frequency. The effect of the reduced FoV on the distribution and average horizontal head movement amplitude was less prominent during walking, and its impact on horizontal eye position distribution was relatively minor during walking and cycling. The influence on the range of vertical eye position was less pronounced in street crossing.

Our findings are partially consistent and partially inconsistent with our hypotheses. The increase in horizontal head movement with larger amplitudes supports the view that individuals compensate for a reduced FoV by performing more and primarily larger head movements. However, we did not observe any alterations in saccadic behaviour under the limited FoV condition. This absence of saccadic change may be attributed to the inherent coupling of saccades with head movements (Freedman 2008), which often automatically triggers saccades prior to a head movement. Consequently, the saccadic frequency and amplitude may remain unaffected by the reduced FoV. Moreover, we only observed a slight overall increase in head movement frequency. Interestingly, both vertical and horizontal ranges in eye position were reduced, suggesting that the saccadic behaviour covered a smaller area within the head reference frame. This may be attributed to the limited amount of additional visual information uptake when scanning a broader area, as the participant’s vision will be occluded by the borders of the reduced FoV. Collectively, these findings seem to indicate that individuals tend to perform more horizontal head movements with larger amplitudes, while reducing the distribution of their eye movements due to the limited FoV of HMD’s. Although individuals may compensate for the limited FoV by performing more head movements with a longer amplitude, obtaining the necessary visual information can still be challenging. At least in virtual reality, individuals may be less able to use peripheral information during ongoing visual search to plan their successive scans (Stein et al. 2024).

Contrary to previous reports that compared scanning behaviour in VR displayed by HMDs to similar real-life situations, we observed a reduction in eye position and no change in saccadic behaviour (Berton et al. 2019, 2020; Drewes et al. 2021). These disparities may be attributed to the activity performed, the virtual environment utilised or other limitations of the HMDs. Our results show that the effect of the reduced FoV on horizontal eye position was less pronounced during walking, the activity examined in prior studies (Berton et al. 2019, 2020; Drewes et al. 2021). Additionally, the virtual environment used, which possibly featured limited details, may have led to less exploration. Lastly, other shortcomings of HMDs may account for the observed disparities. For instance, the peripheral areas of HMD lenses are often blurry, hindering an individuals’ ability to perceive visual information when directing their eye towards these regions. Such limited information intake may, next to the reduced FoV, also result in altered scanning behaviour (Aizenman et al. 2023).

The effect of the reduced FoV varies among mobility activities, likely due to their specific task demands. For instance, peripheral information may be less critical for safe navigation in a shopping street compared to cycling a route or crossing a street. Moreover, head movements are proposed to be essential for safely crossing intersections during cycling and pedestrian street crossing (Hassan et al. 2005; Pundlik et al. 2023), but may be less critical when walking in a shopping street. Additionally, the vertical eye position may assist with foot placement during walking or steering while cycling, but might not facilitate the decision to cross a street. It is, therefore, imperative to consider the task-specific scanning behaviour of each activity and how the technical set-up of HMD’s might influence this behaviour.

The findings in this study suggest caution when translating scanning behaviour outcomes from VR environments displayed through HMDs to real-life settings, since the limited FoV probably does not induce naturalistic eye and head movement patterns. While conducting research in real-life could avoid such concerns, it is inherently less controllable and highly variable, limiting the applicability to various research objectives. Alternatively, simulators featuring an unrestricted FoV could be employed, such as a CAVE system or a multiple screen system. Yet, these alternative systems have their own limitations that could impact scanning behaviour, including a lower sense of presence, the absence of a ground or close-proximity projection of the VR world, the lack of a 360° field of regard, and the absence of stereoscopic vision (Borrego et al. 2016; Schneider et al. 2021; Schneider and Bengler 2020). Therefore, our recommendation is to not avoid the use of HMD’s for investigating scanning behaviour, but rather select the system and environment tailored to the specific task requirements and objectives of the study. Additionally, researchers should be observant of the potential data alterations caused by virtual systems.

Our findings also carry implications for the application of HMD’s within rehabilitation. While virtual environments displayed by HMDs offer a safe and controlled setting to train mobility activities, they currently may foster scanning behaviour that is undesirable in the real-world. For instance, rehabilitation programs focussing on enhancing saccadic behaviour and eye position, such as the compensatory scanning training for people with hemianopia (De Haan et al. 2015; Haan et al. 2016; Pollock et al. 2019) or those with retinitis pigmentosa (Ivanov et al. 2016), should be cautious when incorporating virtual training tasks by using HMDs. Conversely, HMDs can probably be effectively employed to train eccentric viewing in people with macular degeneration or tasks that predominantly demand a central focus, such as cooking. Consequently, to optimally improve rehabilitation programs, it is also crucial to select the training methods that align with the objectives of the rehabilitation program.

While current HMDs are constrained by the limited FoV, the rapid advancements in VR technology may soon overcome this shortcoming. Innovative lenses are currently under development that hold the potential to expand the FoV of HMD’s matching the degree of the human FoV (Nakano et al. 2021; Ratcliff et al. 2020). Notably, specific lenses may even improve the blurriness in the lenses’ periphery (Ratcliff et al. 2020), which also may facilitate more natural scanning behaviour (Aizenman et al. 2023). These ongoing advancements in lens technology hold promise for facilitating natural scanning behaviour in virtual environments displayed by HMD’s.

When interpreting the outcomes of the study the following limitations should be considered. The standardisation of this study is limited as controlling the surroundings was not feasible, since the participants performed the mobility activities in a real-life setting.

While the accuracy of the eye-tracker (mean: 5.3°; standard deviation: 1.6°) was lower than typically achieved in lab-based experiments (0.5°–2.0°), it aligns with the accuracy reported by the manufacturer (4°; Tonsen et al. 2020) and findings from a previous study utilising the Pupil Invisible eye-tracker to investigate gaze behaviour during stair climbing (mean: 3.90°; range: 0.89°–9.64°; Ghiani et al. 2024). Moreover, in our study, we statistically tested for deviations in eye position and saccadic amplitude distributions across different FoV conditions by grouping our data into 10° bins. Since these bins are much wider than the 5.3° measurement accuracy, we expect the accuracy has a minimal impact on our results. It should be noted that the accuracy was probably lower in the peripheral areas (Tonsen et al. 2020), possibly reducing the accuracy of the eye-tracker during street crossing, due to the wider distribution of eye position. Additionally, the head motion data may be less reliable due to the utilisation of an inertial measurement unit without a magnetometer that can decrease the drift in the signal (Ilyas et al. 2016). Nevertheless, we aimed to minimise the influence of drift by classifying each head movement separately and using only this part of the signal to calculate the amplitude.

The data loss due to blinks in our study is relatively high (mean: 20%) compared to most indoor and lab-based studies, but is similar to the outdoor findings of Ghiani et al. (2024). We suspect four possible explanations, namely sunlight interference may have impacted eye recordings, potentially leading to misclassified blinks, fast vertical saccades may have been misclassified as blinks, smaller eye openings, for example due to squinting in response to bright sunlight, may have triggered blink classification, and participants may blink more frequently in outdoor settings. Data loss was particularly high during street crossing (25.9%) and cycling (22.0%) compared to walking (16.6%). This increased data loss may be due to more frequent rapid vertical saccades or greater fluctuations in sunlight exposure, leading to more data being misclassified as blinks. Additionally, during street crossing, we observed that individuals often blink when making quick horizontal head movements, which are common in this situation as people scan both sides of the street. This may have also contributed to the higher data loss due to blinks. As a result, the blink detector algorithm may be less effective for this activity compared to walking or cycling. While the current blink classifying algorithm has its limitations, it is still essential for excluding unreliable data or blinks. Moreover, we do not expect that the data loss will influence the comparison between FoV conditions, as the amount of data loss is similar between those conditions (mean difference is 0.2%).

Next to the blink detector, the saccade classifier has limitations, as it cannot distinguish between saccades and vestibular ocular reflex movements, and small saccades may go undetected during high-noise phases. Additionally, the head movement classifier may have missed small head movements intended to alter visual input. While both saccade and head movements classifiers have limitations, we do not expect that our choice of classifier would significantly impact the observed differences in scanning behaviour across FoV conditions.

The data in this study is limited in both duration and sample size. For detailed information on the data quality, see Tables 4, 5, and 6 in the supplementary material. To assess whether our participants are a representative sample of how individuals scan the environment during mobility activities, we compared our findings to two public datasets (Ghiani et al. 2024; Losing and Hasenjäger 2022; see supplementary material A). It shows that our data on the saccadic behaviour, horizontal head movement behaviour, and horizontal eye position, is similar to the two public datasets. There are some differences in the vertical eye position, showing that participants in our study focus more centrally compared to those in the MMG study (Losing and Hasenjäger 2022) and Ghiani et al. (2024), which can be explained by the differences in setting (walking stairs in Ghiani et al. 2024 for example). Overall our participants appear to show representative scanning behaviour. It should be noted that the duration of the street crossing activity was also much lower compared to the other activities resulting in less recorded data on eye and head movement behaviour. Lastly, it is important to emphasise that this study does not aim to quantify the absolute effect of the reduced FoV on scanning behaviour and it’s advised to not use our finding for such purpose, given the limited data.

Despite its limitations, this study provided valuable insights into how a reduced FoV, featured in current HMDs, affects scanning behaviour in several everyday mobility activities, including cycling, walking and street crossing. These findings are particularly relevant as HMD technology gains popularity in both vision research and rehabilitation.

To conclude, the reduced FoV, as presented in HMDs, probably influences scanning behaviour during mobility activities, with these effects varying between different types of activities. Generally, individuals seem to compensate for the reduced FoV by performing more horizontal head movements with larger amplitudes, while reducing the range in eye position. Consequently, caution is required when translating outcomes on scanning behaviour observed in HMDs to real-life situations. It is crucial for researchers and rehabilitation professionals to select the (virtual) research or training methods that align with the task requirements and objectives of the study or rehabilitation program. Additionally, they should be mindful of the potential alterations in behaviour caused by virtual systems. Future research could investigate whether a reduced field of view changes other eye and head movement patterns, such as the vestibular ocular reflex, smooth pursuit, or gaze direction (combined eye and head rotation).

## Electronic supplementary material

Below is the link to the electronic supplementary material.

Supplementary Material 1

## Figures and Tables

**Fig. 4:**
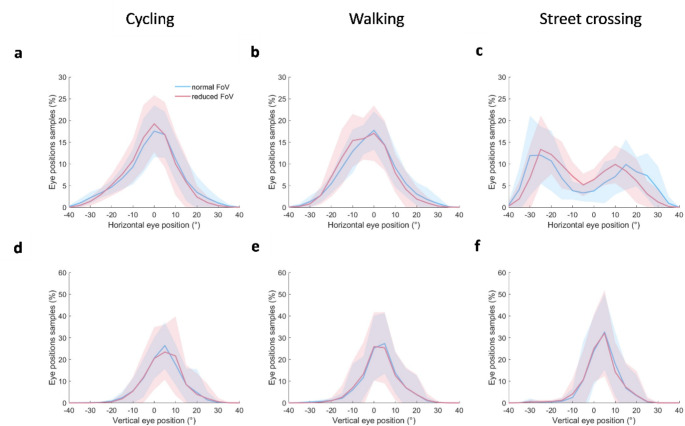
The original distributions of horizontal eye position (**a**–**c**) and vertical eye position (**d**–**f**) for the reduce FoV (r-FoV) condition presented in red and the normal FoV condition (n-FoV) presented in blue per activity; Cycling (left panel), Walking (middle panel), Street crossing (right panel)

## Data Availability

No datasets were generated or analysed during the current study.

## Appendix

See Figs. 4, 5, 6, and 7 and Tables 4, 5, and 6.

**Table 4:** The duration of the three activities per FoV condition and participant

	Normal FoV			Reduced FoV		
	Cycling (min)	Walking (min)	Street crossing (min)	Cycling (min)	Walking (min)	Street crossing (min)
1	5.7	3.9	2.0	5.3	3.9	1.9
2	5.6	4.0	1.3	–	4.0	1.1
3	4.6	4.1	1.5	4.9	4.2	1.3
4	5.8	4.0	1.0	5.2	3.9	2.1
5	4.3	3.5	1.7	4.3	3.5	0.8
6	4.1	3.7	0.7	4.3	3.8	1.3
7	6.1	3.9	1.7	5.2	3.7	1.0
8	4.7	4.0	1.0	5.3	4.4	1.2
9	5.1	4.2	4.2	4.8	–	1.8
10	5.4	5.0	1.6	5.8	5.0	1.9
11	5.0	3.9	0.9	5.2	3.7	1.6
12	6.7	4.6	1.6	6.7	4.1	2.0
13	5.3	4.6	1.8	5.6	4.6	1.7
14	5.6	4.0	1.1	4.9	4.2	2.3
15	7.3	4.6	1.7	6.7	4.4	1.0
16	5.8	4.7	1.1	5.5	4.6	1.4

**Table 5:** The data loss for each participant by activity and FoV condition

	Normal FoV			Reduced FoV		
	Cycling (%)	Walking (%)	Street crossing (%)	Cycling (%)	Walking (%)	Street crossing (%)
1	20.6	14.0	29.6	19.6	16.5	31.0
2	30.0	16.5	26.9	–	14.6	34.7
3	23.4	15.7	23.5	24.8	14.2	21.8
4	13.0	8.5	19.3	15.9	13.3	20.0
5	16.4	11.7	19.4	23.0	14.4	30.2
6	25.0	20.0	49.9	21.7	18.7	29.2
7	11.1	6.7	14.8	10.4	13.7	16.9
8	33.1	27.2	34.3	27.6	22.5	38.0
9	20.6	16.2	15.9	26.9	–	21.4
10	25.8	23.7	31.5	27.1	22.1	24.9
11	30.8	25.5	34.6	34.3	19.8	30.4
12	22.6	19.4	23.6	21.7	16.5	17.9
13	29.6	25.1	32.2	29.3	25.1	27.4
14	21.3	11.2	25.2	21.8	13.6	19.2
15	5.9	7.4	13.9	8.3	4.0	27.5
16	20.2	19.3	27.4	18.7	16.3	17.0

**Table 6:** The accuracy of the eye position data for each calibration validation separately and average over the three calibration validations for each participant

	Accuracy validation 1 (°)	Accuracy validation 2 (°)	Accuracy validation 3 (°)	Accuracy average (°)
1	5.6	3.9	5.7	5.0
2	–	5.2	7.6	6.4
3	2.8	3.2	3.9	3.3
4	3.3	8.1	7.4	6.2
5	4.2	3.0	4.2	3.8
6	7.6	6.2	6.6	6.8
7	4.2	4.2	5.3	4.5
8	4.8	–	7.2	6.0
9	3.6	2.6	4.7	3.6
10	4.7	8.3	5.2	6.1
11	3.4	2.5	2.4	2.8
12	5.0	7.1	–	6.1
13	6.2	5.2	4.6	5.4
14	2.4	4.3	3.2	3.3
15	–	8.0	4.0	6.0
16	–	8.7	8.5	8.6

– indicates missing data due to a failure of the scene video recording or a failure in detecting the QR code

## References

[CR1] Aizenman AM, Koulieris GA, Gibaldi A, Sehgal V, Levi DM, Banks MS (2023) The statistics of eye movements and binocular disparities during VR gaming: implications for headset design. ACM Trans Graphics 42(1). 10.1145/3549529 10.1145/3549529 PMC10139447 37122317

[CR3] Berton F, Olivier AH, Bruneau J, Hoyet L, Pettre J (2019) Studying gaze behaviour during collision avoidance with a virtual walker: Influence of the virtual reality setup. In: 26th IEEE conference on virtual reality and 3D user interfaces, VR 2019 - proceedings, 717–725. 10.1109/VR.2019.8798204

[CR2] Berton F, Hoyet L, Olivier A-H, Bruneau J, Le Meur O, Pettré J, Eì E, Olivier A-H, ´, Pettre J (2020) Eye-gaze activity in crowds: impact of virtual reality and density. https://hal.archives-ouvertes.fr/hal-02544516

[CR4] Borrego A, Latorre J, Llorens R, Alcañiz M, Noé E (2016) Feasibility of a walking virtual reality system for rehabilitation: objective and subjective parameters. J Neuroeng Rehabil 13(1). 10.1186/s12984-016-0174-1 10.1186/s12984-016-0174-1 PMC4977644 27503112

[CR5] Clay V, König P, König S (2019) Eye tracking in virtual reality. J Eye Mov Res 12(1). 10.16910/jemr.12.1.3 10.16910/jemr.12.1.3 PMC7903250 33828721

[CR6] De Haan GA, Melis-Dankers BJM, Brouwer WH, Tucha O, Heutink J (2015) The effects of compensatory scanning training on mobility in patients with homonymous visual field defects: a randomized controlled trial. PLoS ONE 10(8). 10.1371/journal.pone.0134459 10.1371/journal.pone.0134459 PMC4537273 26275160

[CR7] De Haan GA, Melis-Dankers BJM, Brouwer WH, Tucha O, Heutink J (2016) The effects of compensatory scanning training on mobility in patients with homonymous visual field defects: further support, predictive variables and follow-up. PLoS ONE 11(12). 10.1371/journal.pone.0166310 10.1371/journal.pone.0166310 PMC5147814 27935973

[CR8] Drewes J, Feder S, Einhäuser W (2021) Gaze during locomotion in virtual reality and the real world. Front NeuroSci 15. 10.3389/fnins.2021.656913 10.3389/fnins.2021.656913 PMC8180583 34108857

[CR9] Finley JM, Gotsis M, Lympouridis V, Jain S, Kim A, Fisher BE (2021) Design and development of a virtual reality-based mobility training game for people with Parkinson’s disease. Front Neurol 11. 10.3389/fneur.2020.577713 10.3389/fneur.2020.577713 PMC7843522 33519665

[CR10] Foulsham T (2015) Eye movements and their functions in everyday tasks. Eye (Basingstoke) 29(2):196–199. 10.1038/eye.2014.275 10.1038/eye.2014.275 PMC4330286 25397783

[CR11] Freedman EG (2008) Coordination of the eyes and head during visual orienting. In: Experimental brain research, vol 190, Issue 4. pp 369–387. 10.1007/s00221-008-1504-8 10.1007/s00221-008-1504-8 PMC2605952 18704387

[CR12] Gestefeld B, Koopman J, Vrijling A, Cornelissen FW, de Haan G (2020) Eye tracking and virtual reality in the rehabilitation of mobility of hemianopia patients: a user experience study. Int J Orientat Mobil 11(1):7–19. 10.21307/vri-2020-002

[CR13] Ghiani A, Mann D, Brenner E (2024) Methods matter: exploring how expectations influence common actions. iScience 27(3):109076. 10.1016/j.isci.2024.109076 38361615 10.1016/j.isci.2024.109076 PMC10867666

[CR14] Hassan SE, Geruschat DR, Turano KA (2005) Head movements while crossing streets: effect of vision impairment 15630400

[CR15] Hooge I, Camps G (2013) Scan path entropy and arrow plots: capturing scanning behavior of multiple observers. Front Psychol 4(12). 10.3389/fpsyg.2013.00996 10.3389/fpsyg.2013.00996 PMC3872074 24399993

[CR17] Ilyas M, Cho K, Baeg SH, Park S (2016) Drift reduction in pedestrian navigation system by exploiting motion constraints and magnetic field. Sens (Switzerland) 16(9). 10.3390/s16091455 10.3390/s16091455 PMC5038733 27618056

[CR18] Ivanov IV, Mackeben M, Vollmer A, Martus P, Nguyen NX, Trauzettel-Klosinski S (2016) Eye movement training and suggested gaze strategies in tunnel vision - a randomized and controlled pilot study. PLoS ONE 11(6). 10.1371/journal.pone.0157825 10.1371/journal.pone.0157825 PMC4924791 27351629

[CR16] Losing V, Hasenjäger MA, Multi-Modal (2022) Gait database of natural everyday-walk in an urban environment. Sci Data 9:473. 10.1038/s41597-022-01580-3 35922448 10.1038/s41597-022-01580-3 PMC9349224

[CR19] Nakano K, Isoyama N, Monteiro D, Sakata N, Kiyokawa K, Narumi T (2021) Head-Mounted display with increased downward field of view improves presence and sense of self-location. IEEE Trans Vis Comput Graph 27(11):4204–4214. 10.1109/TVCG.2021.3106513 34449388 10.1109/TVCG.2021.3106513

[CR20] Nazemi M, van Eggermond MAB, Erath A, Schaffner D, Joos M, Axhausen KW (2021) Studying bicyclists’ perceived level of safety using a bicycle simulator combined with immersive virtual reality. Accid Anal Prev 151. 10.1016/j.aap.2020.105943 10.1016/j.aap.2020.105943 33370601

[CR21] Pala P, Cavallo V, Dang NT, Granié MA, Schneider S, Maruhn P, Bengler K (2021) Analysis of street-crossing behavior: comparing a CAVE simulator and a head-mounted display among younger and older adults. Accid Anal Prev 152. 10.1016/j.aap.2021.106004 10.1016/j.aap.2021.106004 33540347

[CR22] Pollock A, Hazelton C, Rowe FJ, Jonuscheit S, Kernohan A, Angilley J, Henderson CA, Langhorne P, Campbell P (2019) Interventions for visual field defects in people with stroke. In: Cochrane database of systematic reviews, vol 2019, Issue 5. Wiley. 10.1002/14651858.CD008388.pub3 10.1002/14651858.CD008388.pub3 PMC6532331 31120142

[CR23] Pundlik S, Tomasi M, Houston KE, Kumar A, Shivshanker P, Bowers AR, Peli E, Luo G (2023) Gaze scanning at street crossings by pedestrians with homonymous hemianopia with and without hemispatial neglect. Investig Ophthalmol Vis Sci 64. 10.1167/iovs.64.14.26 10.1167/iovs.64.14.26 PMC10680492 37975848

[CR24] Ratcliff J, Supikov A, Alfaro S, Azuma R (2020) ThinVR: heterogeneous microlens arrays for compact, 180 degree FOV VR near-eye displays. IEEE Trans Vis Comput Graph 26(5):1981–1990. 10.1109/TVCG.2020.2973064 32070971 10.1109/TVCG.2020.2973064

[CR25] Sakhare AR, Yang V, Stradford J, Tsang I, Ravichandran R, Pa J (2019) Cycling and Spatial navigation in an enriched, immersive 3D virtual park environment: a feasibility study in younger and older adults. Front Aging Neurosci 11. 10.3389/fnagi.2019.00218 10.3389/fnagi.2019.00218 PMC6706817 31474851

[CR26] Schneider S, Bengler K (2020) Virtually the same? Analysing pedestrian behaviour by means of virtual reality. Transp Res Part F Traffic Psychol Behav 68:231–256. 10.1016/j.trf.2019.11.005

[CR27] Schneider S, Maruhn P, Dang N-T, Pala P, Cavallo V (2021) Pedestrian crossing decisions in virtual environments: behavioral validity in CAVEs and head-mounted displays 10.1177/0018720820987446 33529060

[CR28] Schwebel DC, Gaines J, Severson J (2008) Validation of virtual reality as a tool to understand and prevent child pedestrian injury. Accid Anal Prev 40(4):1394–1400. 10.1016/j.aap.2008.03.005 18606271 10.1016/j.aap.2008.03.005

[CR29] Stein N, Watson T, Lappe M et al (2024) Eye and head movements in visual search in the extended field of view. Sci Rep 14:8907. 10.1038/s41598-024-59657-5 38632334 10.1038/s41598-024-59657-5 PMC11023950

[CR30] Tonsen M, Baumann CK, Dierkes K (2020) A high-level description and performance evaluation of pupil invisible. http://arxiv.org/abs/2009.00508

[CR31] van Paridon K, Timmis MA, Sadeghi Esfahlani S (2021) Development and evaluation of a virtual environment to assess cycling hazard perception skills. Sensors 21(16). 10.3390/s21165499 10.3390/s21165499 PMC8402292 34450941

[CR32] Zeuwts LHRH, Iliano E, Smith M, Deconinck F, Lenoir M (2021) Mental fatigue delays visual search behaviour in young cyclists when negotiating complex traffic situations: A study in virtual reality. Accid Anal Prev 161. 10.1016/j.aap.2021.106387 10.1016/j.aap.2021.106387 34492561

