# Supplementary material for: Reduced field of view alters scanning behaviour
Source: Virtual Real. 2025 Mar 22;29(2):55. doi: 10.1007/s10055-025-01125-0 (PMC11928371; doi:10.1007/s10055-025-01125-0)
Supplement: Supplementary file 1 — Supplementary Material 1 [file 10055_2025_1125_MOESM1_ESM.docx]

**Supplementary Material: Comparing eye- and head-tracking data to similar public available data**

One limitation of our study is the relatively low amount of data in terms of both duration and number of participants. To determine if our sample is representative, we compared our findings to publicly available datasets in which participants performed similar tasks.

**Dataset 1: Losing & Hasenjäger, 2022**

The first dataset we used for comparison is “A Multi-Modal Gait Database of Natural Everyday-Walk in an Urban Environment” (Losing & Hasenjäger, 2022). This study also employed the Pupil Invisible eye-tracker (60 Hz) to collect data on gaze behavior, eliminating potential variability due to differences in eye-tracker technology. This data set did not include the IMU data of the Pupil Invisible.

In this dataset, participants (N=20, mean age = 36.8 ± 10.75 years, 25% female) walked three different routes near a suburban train station. Courses A and B, each approximately 500 meters, included level-ground walking, stair ascent and descent, and ramp navigation. Course C, approximately 200 meters, included level-ground walking and stepping on and off a curb. The average duration was 11.7 minutes for Course A, 9.9 minutes for Course B, and 6.5 minutes for Course C.

**Dataset 2: Ghiani et al., 2024**

The second dataset, part of the experiment reported by Ghiani et al. (2024), includes recordings of eye position and head rotation for six participants walking toward an open square. Stair-climbing data were excluded from this comparison. This study used the Pupil Invisible eye-tracker at a sampling rate of 200 Hz. The average recording duration was 8.0 minutes.

**Our Study**

We compared these datasets with our data on participants walking in a shopping street with a normal field of view, aiming to assess consistency in scanning behavior across different settings.


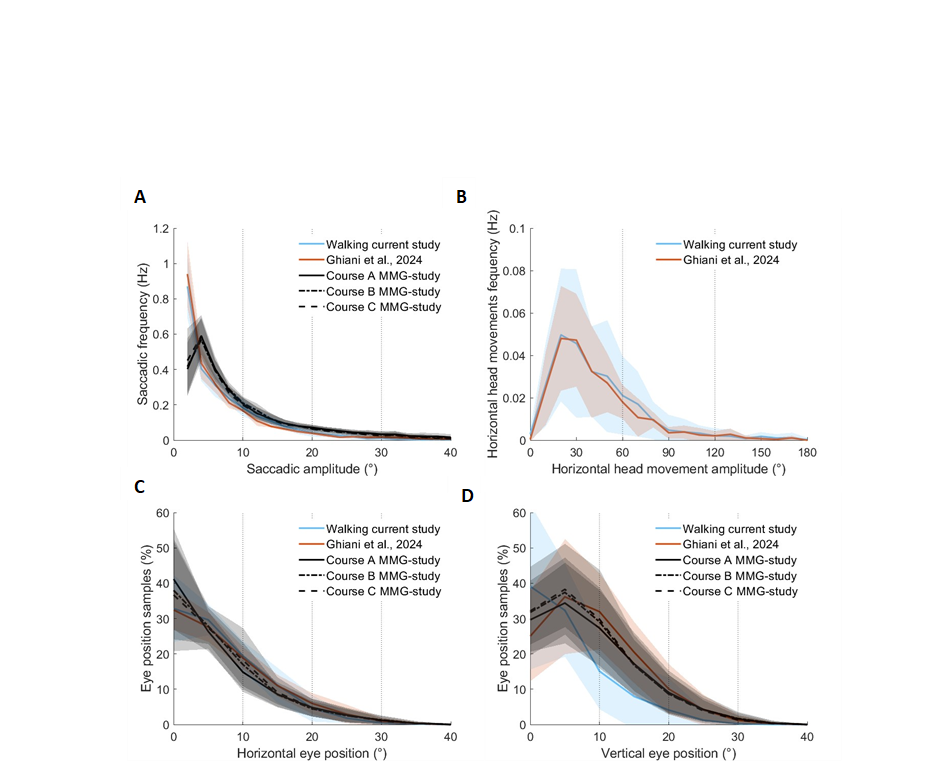


*Figure S1.* **Comparison of eye and movement behaviour when walking with a normal field of view to Ghiani et al. (2024) and the MMG database.** Panel a shows the distribution of saccadic frequency, panel b shows the distribution of horizontal head movement frequency, panel c shows the distribution of horizontal gaze position, and panel d shows the distribution of vertical eye position of walking with a normal field of view (blue), Course A of the Multi-Modal Gait (MMG) study (black straight line), Course B of the Multi-Modal Gait (MMG) study (black semi-dotted line), and Course C of the Multi-Modal Gait (MMG) study (black dotted line).

Figure S1 shows the distributions of saccadic frequency, horizontal eye position, and vertical eye position across the datasets. There is a slight deviation in saccadic frequency for small-amplitude saccades (approximately 1-5 degrees; Fig. S1a), likely due to the lower sampling rate of the MMG dataset (60 Hz) compared to our study and Ghiani et al. (2024) (200 Hz), which may affect the detection accuracy of small saccades.

Additionally, we observed a difference in vertical eye position across datasets. Participants in our study, who walked with a normal field of view, tended to direct their gaze more centrally (0-10 degrees) compared to participants in the MMG study and Ghiani et al. (2024), who directed their gaze more toward peripheral areas (10-30 degrees).

The horizontal eye position, and the horizontal head movement amplitude distribution did not deviate from the two other datasets.

In conclusion, the distributions of saccadic frequency and horizontal eye position appear representative of scanning behavior during walking. While there is a discrepancy in the frequency of small saccades, this likely stems from the lower sampling rate of the MMG dataset. It is less clear whether our vertical eye position distribution is fully representative of typical scanning behavior during walking, as participants in our study tended to focus more centrally compared to those in the MMG study and Ghiani et al. (2024).
